# Biology and Endodontics: Thinking Outside the Box

**DOI:** 10.7759/cureus.25277

**Published:** 2022-05-24

**Authors:** Turki Y Alhazzazi

**Affiliations:** 1 Oral Biology, Faculty of Dentistry, King Abdulaziz University, Jeddah, SAU

**Keywords:** case report, ebp, ebd, endodontics, biology

## Abstract

Combining evidence-based dentistry (EBD) with years of clinician experience should have a great impact on treatment planning and decision-making for achieving the best treatment outcomes, thus warranting patient satisfaction. In addition, understanding and appreciating the role of biology on the reliance on and progress of treatment healing is also a crucial element that clinicians should always keep in mind in their dental practice. This study demonstrates that clinicians should always rely on their own clinical and radiographical test results for evaluation and judgment of any clinical situation before presenting and proceeding with any dental treatment for their patients.

## Introduction

Evidence-based dentistry (EBD) and evidence-based practice (EBP) are terms related to applying the best available evidence gained from scientific research and methodology to form the basis of clinical decision-making [1-3]. However, biology sometimes has its ways of surprising us when it comes to treatment outcomes. Keeping such surprises in mind, especially when combining EBD/EBP with clinician years of experience, may crucially impact our daily dental practice decision-making when it comes to treatment planning and evaluating treatment outcomes. This study demonstrates that clinicians should always rely on their own clinical and radiographical test results for evaluation and judgment of any clinical situation before presenting and proceeding with any dental treatment for their patients.

## Case presentation

**Figure 1:**
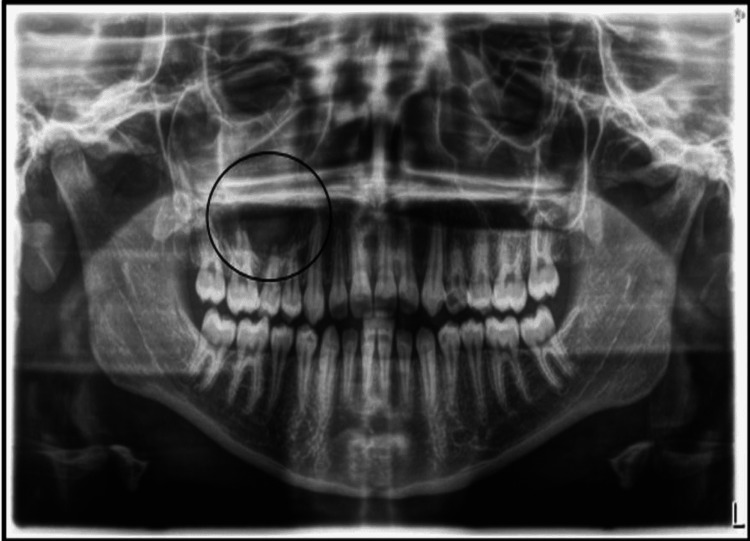
The preoperative panoramic radiograph that was presented by the patient, during the initial consultation visit, showing a poor quality image that masks the presence of the small primary remaining root in the upper right side of the maxilla (circle).

A 14-year-old girl presented to our clinic in early May 2021, with the chief complaint of a swollen face one week ago. She was given antibiotics and told that she needed root canal treatment (RCT) for several teeth that were causing the swelling. In addition, the patient was presented with a medical report and panoramic radiograph by her previous dentist, stating that she needed RCT for teeth numbers #16, #15, and #14 and that due to the size of the lesion, she would need to undergo a post-root canal surgical procedure to remove the large lesion associated with these teeth at her upper right maxilla (Figure 1).

Her past medical history was insignificant, as she had no underlying medical issues or allergies. Her vital signs were within normal range: heart rate of 65 bpm and blood pressure of 110/75 mmHg. The patient presented with no fever.

Clinical examination revealed that her oral hygiene was fair; several small to moderate carious teeth were noticed, especially at teeth numbers #26 and #24. In addition, teeth numbers #16, #15, and #14 were all sound with no associated cavities or fillings. The offending area seemed very tender to palpation only at the area apical to tooth number #15 and also sensitive to percussion test, although there was no longer any sign of swelling anywhere in the area.

**Figure 2:**
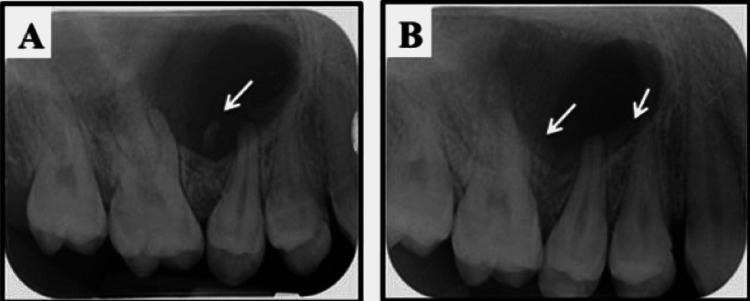
(A) Preoperative periapical radiograph clearly showing the small primary remaining root between the roots of teeth number 16 and 15 (arrow) and the large lesion causing diversion to the number #16 and #15 roots. (B) Postoperative periapical radiograph clearly showing the successful removal of the small primary remaining root without radiographically affecting any of the associated teeth roots. Bony deposition started at the surgical area (arrows) (three-month follow-up).

**Figure 3:**
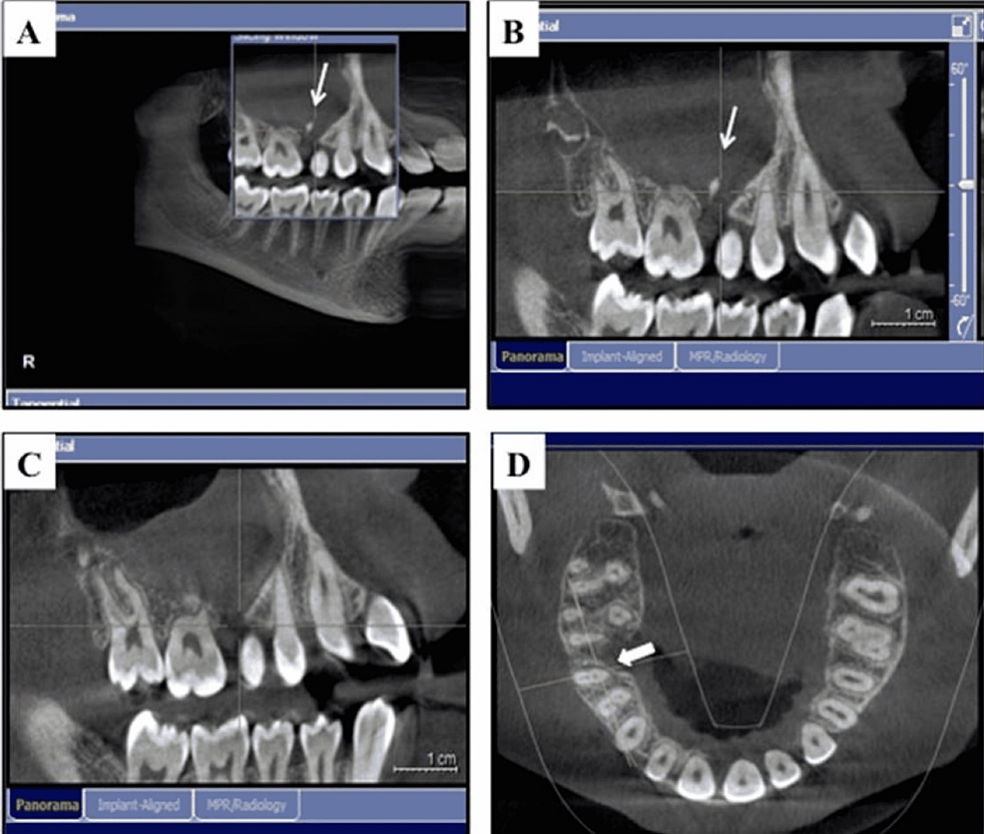
(A) A preoperative representative section of the cone-beam computed tomography (CBCT) that was taken during the initial consultation to evaluate the size and extension of the lesion and clearly showing the small primary remaining root between the roots of teeth 16 and 15. (B) A close-up view. (C) A postoperative representative section (three-month follow-up) of the CBCT demonstrating the successful removal of the primary remaining root. (D) A postoperative representative section showing the bony deposition starting at the surgical area (arrows) (three-month follow-up).

Full diagnostic radiographic records were obtained for the patient, including left and right bitewings and periapical radiographs for teeth numbers #16, #15, #14, and #24 (to evaluate the deep caries lesion) (Figure 2). Finally, a cone-beam computed tomography (CBCT) was taken to evaluate the size and the extension of the lesion (Figure 3). Radiographic examination revealed the presence of a small primary remaining root between teeth numbers #16 and #15, which are the likely cause of this massive lesion and also of a severe diversion between the roots (Figure 2A, Figures 3A-3B). This important finding was apparently misdiagnosed by her previous dentist, who evaluated the cause of her complaint using only the panoramic radiograph, which appeared to be of poor quality, as seen in Figure 1, and was not able to clearly show the small primary remaining root between teeth numbers #16 and #15. In addition, it seems that the previous dentist did not perform any vitality tests for the offending teeth. We performed cold (Endo-Ice, 1,1,1,2 Tetrafluoroethene; Hygenic Corp, Akron, OH) and electric pulp tests (EPT) for all offending teeth and compared the results using opposing contralateral teeth as controls, with the exception of tooth number 24, which was excluded as a control due to the presence of deep caries; thus, tooth number 45 was used instead. All offending teeth were vital and responded within the normal range to both cold (~3-5 seconds with quick recovery and no sign of any lingering pain) and EPT tests compared to controls. Moreover, tooth number 15 was the only one tender to persuasion and palpation. After a clinical and radiographic evaluation, the endodontic diagnosis for teeth numbers #16, #15, and #14 was normal pulp with normal periapical tissues, normal pulp with symptomatic apical periodontitis, and normal pulp with normal periapical tissues, respectively. 

With these clinical and radiographical findings, a treatment plan was discussed with the patient and her parents as follows: Considering the age of the patient and that the cause of the large lesion and recent swelling was not the teeth in question, RCT was not recommended. Thus, there was no rationale for jumping to the conclusion that root canals were needed before attempting the surgical procedure to remove the lesion and the associated primary remaining root as shown in the periapical radiographs and CBCT images, which appeared to be the participating cause (Figure 2A, Figures 3A-3B). During the surgery, a biopsy would be taken to roll out any unexpected pathological cause, and follow-up visits would be required periodically for several years to ensure the progress of the healing process and to assess the vitality of the offending teeth post-surgical intervention, in case innervation/vitality was affected after the removal of this large lesion in one or more of these teeth.

The patient’s parents agreed to this course of treatment and requested that their daughter be referred to an expert maxillofacial surgeon at King Abdulaziz University Dental Hospital (UDH), Jeddah, Saudi Arabia. 

**Figure 4:**
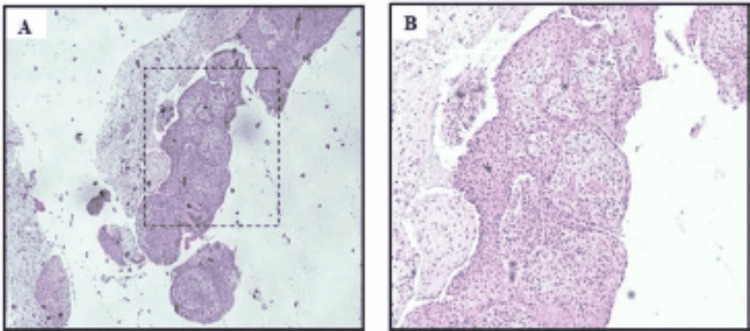
(A) A representative histological picture stained with H&E showing a lining epithelium and connective tissue (CT) stroma. (B) A higher magnification view (20x) showing the CT stroma presented with heavy infiltration of chronic inflammatory cells (lymphocytes and plasma cells), in addition to small blood vessels that are lined by a single layer of endothelial cells. The lining epithelium did not show any dysplastic or atypical cells.

Due to the young age of the patient and the size of the lesion, the maxillofacial surgeon, after discussing the treatment plan with the parents, decided to conduct the surgery under general anesthesia. A biopsy was obtained during the procedure to confirm the diagnosis as part of the normal surgical protocol at UDH. After surgery, the patient was prescribed antibiotics-1g augment BID for seven days and 400 mg Ibuprofen PRN-and scheduled for a follow-up visit after seven days. The biopsy specimen revealed the presence of stratified squamous epithelial lining cells supported by connective tissue (CT) stroma. The CT stroma showed heavy infiltration of chronic inflammatory cells (lymphocytes and plasma cells), in addition to small blood vessels that were lined by a single layer of endothelial cells (Figure 4). The lining epithelium did not show any dysplastic or atypical cells (Figure 4B). Thus, the tentative diagnosis was a choric inflammatory odontogenic cyst. Based on the clinical correlation that showed the association of the lesion with the primary remaining root, the diagnosis most probably was an inflamed radicular cyst. Due to the patient’s schedule, she was seen by the surgeon about 10 days later for post-surgical evaluation. Clinical and radiographical evaluation was promising as the patient presented with very satisfactory post-surgical healing and was happy and pain-free.

The patient was also seen in our dental clinic at three and six months post-surgery. Intraoral examination showed excellent healing, and good gingival margin contouring was achieved. Periapical radiographs and CBCT were also taken to evaluate post-surgical images (Figure 2B, Figures 3C-3D). They showed no sign of the primary remaining root and also showed the intact roots of the associated post-surgical teeth (Figure 2B, Figure 3C). In addition, bony deposition started in the surgical area (Figure 2B, Figure 3D). Interestingly, teeth numbers #16, #15, and #14 all remained vital and responded normally to both cold and EPT tests, with comparable readings to those recorded in the pre-surgical evaluation visit. Moreover, none of the mentioned teeth showed signs of tenderness to palpation or persuasion. The patient was asked to come for another follow-up visit after three months and to keep in touch if any changes or pain occurred before the next evaluation period. Thus, this case report was carried out from May 2021 to December 2021.

## Discussion

Proper diagnosis is the first step toward any successful dental treatment outcome. Several misdiagnosed cases of non-endodontic origin have been reported in the literature, with results ranging from definite diagnosis of less aggressive lesions such as periodontal abscess and central giant cell granuloma (CGCG) to more aggressive types such as odontogenic keratocysts or even malignancies as squamous cell carcinoma [4-7]. Fortunately, in our case report, the pathology results of the biopsy were reported as an inflamed odontogenic cyst representing an inflammatory radicular cyst due to its association with the primary remaining root, which was misdiagnosed by the patient’s previous dentist.

A previous study conducted by our group contributed to the knowledge and behavior of general dental practitioners (GDPs) regarding following the proper standards of care while performing RCT in Saudi Arabia [8]. The results revealed that the majority (70.3%) of the GDPs would take one or more radiographs, depending on the treated case [8]. As demonstrated in this report, it is obvious that the previous dentist relied only on the panoramic radiograph, in which he focused on the large lesion and missed identifying the cause of the problem, the small primary remaining root, perhaps in part due to the poor quality of the radiograph or his lack of experience (Figure 1). In addition, our previous study revealed that only 42% of the GDPs who participated used the cold test as a tool to confirm endodontic etiology, and 55% believe that pain on the percussion test is a reliable method to draw a conclusion of confirmed endodontic etiology [8]. In this report, it is clear that the previous dentist did not perform any vitality tests on the involved teeth. This was also confirmed by the parents’ own words when they watched those tests being performed in our clinic. Thus, the girl’s previous dentist simply assumed that the lesion was caused by one or more of those teeth and that after surgical intervention they would all lose vitality, leading him to suggest performing RCTs for all these teeth in advance. From our clinic’s point of view, this is an outrageous extreme of malpractice, especially considering the age of the patient. Indeed, all of her teeth are still vital post-surgically, as confirmed by our six-month clinical and radiographical evaluation follow-up visits. In our study, cold and EPT were used together, along with the appreciated controls as mentioned previously, to improve the diagnostic sensitivity and reliability of the tests [9-11]. The diagnostic tests and the endodontic diagnosis terminology used in this study were aligned with the American Association of Endodontics (AAE) guidelines [12]. 

Furthermore, performing CBCT in such cases is important and advantageous in evaluating the size and extension of such large lesions before attempting surgical intervention [13,14]. Regarding the retained teeth' vitality, it is important to understand that these sets of teeth receive their innervation and blood supply from a dense bundle of main neurovascular and anastomosis branches [15,16]. In addition, it is important to understand and appreciate that the patient’s young age in this case report was an important factor in the clinical decision-making and treatment planning. Thus, younger age is associated with a robust regenerative ability compared to older age, which would be a positive pivotal player regarding retaining future teeth vitality and ensuring normal bone healing [17]. However, periodic long follow-up visits are still required to monitor the bone’s reparative process, ensure the persistence of vitality of all associated teeth, and finally to exclude any underlying undiagnosed pathological condition due to section cutting and processing.

Furthermore, GDPs seem to undervalue the importance of long-term follow-up of their dental treatment cases. These follow-up visits ensure the proper evaluation of true success and failure of any given treatment outcomes, as patients consider treatment to be successful if there is no pain, but GDPs should understand that is not always the case. Thus, chronic failed RCTs or slowly growing lesions can be painless for years until they explode after causing massive bone loss and distraction, hence heavily affecting treatment outcomes and prognosis. 

In our 2019 study, only 28.7% of the participants reported following up on their RCT cases after treatment [8]. This underscores an important issue in our dental community, as periodic follow-up is considered mandatory, especially in cases similar to our case report, to record both vitality and bone-healing progress and roll out any unexpected undiagnosed pathological condition that might participate in relapse or recurrence down the road, especially in the case of a young patient. 

## Conclusions

Dentists in all specialties should rely on their own clinical and radiographical test results to reach their own diagnosis before presenting and proceeding with any dental treatment for their patients. Combining EBD/EBP with years of clinician experience and properly understanding and appreciating the role of biology, in the reliability and progress of treatment healing, will play a crucial role when it comes to treatment planning and decision-making. Hence, thinking outside the box is always important when facing such cases. Keeping up with the challenge when planning treatment for such cases may be worth trusting the magic of biology, especially in young patients, who harbor a robust and regenerative healing ability.
